# Summary cortisol reactivity indicators: Interrelations and meaning

**DOI:** 10.1016/j.ynstr.2015.04.002

**Published:** 2015-04-30

**Authors:** Jennifer E. Khoury, Andrea Gonzalez, Robert D. Levitan, Jens C. Pruessner, Kevin Chopra, Vincenzo Santo Basile, Mario Masellis, Alasdair Goodwill, Leslie Atkinson

**Affiliations:** a Department of Psychology, Ryerson University, Canada; b Department of Psychiatry and Behavioural Neurosciences, McMaster University, Canada; c Department of Psychiatry, University of Toronto, Canada; d Department of Psychiatry, McGill University, Canada; e Department of Neurology, Sunnybrook Health Sciences Centre, Canada

**Keywords:** Cortisol, Biometrics, Principal component analysis

## Abstract

Research on the hypothalamic pituitary adrenal (HPA) axis has involved a proliferation of cortisol indices. We surveyed recently published HPA-related articles and identified 15 such indices. We sought to clarify their biometric properties, specifically, how they interrelate and what they mean, because such information is rarely offered in the articles themselves. In the present article, the primary samples consist of community mothers and their infants (N = 297), who participated in two challenges, the Toy Frustration Paradigm and the Strange Situation Procedure. We sought to cross-validate findings from each of these samples against the other, and also against a clinically depressed sample (N = 48) and a sample of healthy older adults (N = 51) who participated in the Trier Social Stress Test. Cortisol was collected from all participants once before and twice after the challenges. These heterogenous samples were chosen to obtain the greatest possible range in cortisol levels and stress response regulation. Using these data, we computed the 15 summary cortisol indices identified in our literature survey. We assessed inter-relations amongst indices and determined their underlying dimensions via principal component analysis (PCA). The PCAs consistently extracted two components, accounting for 79%–93% of the variance. These components represent “total cortisol production” and “change in cortisol levels.” The components were highly congruent across challenge, time, and sample. High variable loadings and explained factor variance suggest that all indices represent their underlying dimensions very well. Thus the abundance of summary cortisol indices currently represented in the literature appears superfluous.

Stress physiology plays a role in a variety of human disease processes, physical and psychological (Gallagher et al., 2009, Reynolds, 2013, Turner-Cobb, 2005). It is likely programmed prenatally, in the early postnatal period (Lesage et al., 2006, Levine, 2005, Meaney and Szyf, 2005), and continues to develop throughout childhood and adolescence (see Gunnar and Vazquez, 2006 for a review). Central to stress physiology is the hypothalamic-pituitary-adrenal (HPA) axis with its end-product, cortisol. The HPA axis is of increasing interest in both the developmental (Gunnar et al., 2009) and adult (Staufenbiel et al., 2012) literature as these pertain to healthy (Dickerson and Kemeny, 2004) and clinical (O'Keane et al., 2012) populations.

Perhaps because of this broad interest, the study of HPA function has been accompanied by a proliferation of cortisol indices (e.g., baseline, area under the curve with respect to ground (AUCG), percent change from baseline to peak, etc.). In the current manuscript, we aim to assess the biometric properties of indices commonly used in the psychosocial cortisol challenge literature in an attempt to understand their shared and unique meanings.

Uncertainty regarding the selection of the most suitable cortisol indices has long been a part of the literature. Thus, Pruessner and colleagues recommended the use of both AUC_G_, area under the curve with respect to ground, and AUC_I_, area under the curve with respect to increase, to alleviate difficulties in analyzing datasets containing repeated measures of cortisol (Pruessner et al., 2003). Emphasizing that AUC_G_ and AUC_I_ capture different aspects of the cortisol response, Pruessner et al. (2003) recommended using both measures in conjunction. Despite this notion, AUC_I_ is rarely used in the literature (Fekedulegn et al., 2007), a fact attributed to widespread misunderstanding of what AUC_I_ captures — cortisol change (i.e., increase or decrease), rather than increase alone — and a failure to appreciate the index's biometric properties, including how it relates to other cortisol indices (Fekedulegn et al., 2007). Fekudelegn et al. used principal component analyses to better delineate the biometric properties of AUC_I_ in the context of diurnal cortisol change and response to awakening (Fekedulegn et al., 2007).

Researchers have also noted that the baseline cortisol level is often incorrectly used as a nonspecific proxy for the anticipatory stress response (Engert et al., 2013). Other researchers continue to emphasize the need for consistency in cortisol research, in order to draw comparisons across studies (Dickerson and Kemeny, 2004). For instance, some authors question the appropriateness of point estimates in assessing mother-infant cortisol attunement, suggesting that trajectories may be better suited for capturing dyadic fluctuations in cortisol (Laurent et al., 2011). Furthermore, others point to difficulties in drawing unequivocal conclusions across a proliferation of cortisol indices (Atkinson et al., 2013), particularly in light of the many sources of variation in acute stress responses (e.g., individual differences, features of the stressor, aspects of the collection procedure, etc.) (Dickerson and Kemeny, 2004, Foley and Kirschaum, 2010). Rather than focusing on the association among various cortisol indices with behavioral or health variables, in the current study, we focus on the interrelations amongst cortisol indices and their underlying structure.

For that purpose, we identified several of the cortisol indices that are commonly used in the literature, in order to derive a reasonable sampling of indices for use in the current study. We restricted ourselves to articles published between January 2011 and June 2013 in six journals (Developmental Psychobiology, Early Human Development, Journal of Biological Psychiatry, Psychoneuroendocrinology, Psychosomatic Medicine, and Stress). We chose those journals as they regularly feature research on relations between HPA activity and behavioral/psychiatric function. Rather than being a systematic review, the aim was to generate a comprehensive list of commonly used point indices of cortisol. We reviewed 219 articles that measured cortisol in the context of some form of challenge, with a total of 15 indices of cortisol.

In total, the articles reviewed thus incorporated 15 unique indices of cortisol (see Table 1 for list of indices with definitions). From the original literature search, the majority of the studies did not provide a rationale for use of a particular index, and only 16 provided a citation for the chosen index. Moreover, among these studies, there were instances when the cortisol index selected was not entirely supported by the citation provided. For example, one study used AUC_G_ but inaccurately defined it as assessing cortisol reactivity (Pruessner et al., 2003). Overall, only three studies provided justification for why a specific index was chosen over other indices.

Several studies used different formulas to calculate identically labeled indices. For example, thirteen studies measured “cortisol reactivity,” either by calculating the difference between the baseline and middle sample, last sample, or peak sample. We consider this to be problematic as the use of multiple, varying formulas to then calculate apparently identical indices of ‘cortisol reactivity’ confounds the meaning of results, undermines the possibility of comparison across studies, and makes it difficult to draw valid conclusions for the field a whole. Finally, some studies use different nomenclature but equivalent formulas to assess identical constructs (e.g., AUC_I_ and AUC_AB_, see Table 1).

The purpose of the present study is thus to clarify the meaning of the point indices identified in the aforementioned survey (Table 1) by (a) formally assessing the intercorrelations amongst them and (b) characterizing their underlying dimensions via principal components analysis. We assessed congruence across two primary samples, consisting of mothers and their infants, as well as two validation samples, consisting of clinically depressed adults and healthy older adults. These samples were selected to generalize findings across development (i.e., infants and older adults), as well as across healthy (i.e., mothers, older adults) and clinical (i.e., depressed) samples. In addition, we assessed congruence of findings for these samples across time using three challenges known to have differential impact on the adrenocortical function of participants.

## 1 Method and material

### 1.1 Ethics statement

This study was approved by the Ryerson University Research Ethics Board, the Centre for Addiction and Mental Health Research Ethics Board, and the Institutional Review Board of the Faculty of Medicine of McGill University. The samples of mothers, older adults, and depressed participants provided written, informed consent to participate in the study. We obtained written, informed consent from mothers with respect to their infants' participation. These consent procedures were approved by the respective Research Ethics Boards.

### 1.2 Participants

#### 1.2.1 Samples of mothers and infants

A community sample of 297 mother-infant dyads (52.2% female) participated in home and laboratory visits (Atkinson et al., 2013). During the home visit, mothers were a mean of 33.43 years (*SD* = 4.54) and infants were a mean of 15.98 months (*SD* = 1.37). During the laboratory visit, approximately one month later, mothers were a mean age of 33.75 years (*SD* = 4.42) and infants were a mean of 17.26 months (*SD* = 1.92). Participants were primarily Caucasian (76.0%) and in spousal relationships (82.0%). Almost half (46.2%) were university educated and median family income was between $114,000–150,000 Canadian. This is a demographically low-risk sample.

#### 1.2.2 Sample of depressed adults

A sample of 48 depressed adults participated in a laboratory visit during which they were exposed to the Trier Social Stress Test (Chopra et al., 2009). All patients met criteria for chronic major depressive disorder (defined as experiencing a non-remitting major depressive episode for at least a 2 year period) based on the Structured Clinical Interview for DSM-IV TR criteria) and had a score of at least 18 on the Hamilton Depression Rating Scale (Williams et al., 1988). This sample was also screened for cognitive impairment. The depressed sample ranged from 23 to 55 years old (*M* = 41.62, *SD* = 1.12), with 41.5% males.

#### 1.2.3 Sample of older adults

A sample of 51 older adults was also exposed to the Trier Social Stress Test. Older adults did not have a history of Axis I disorders (according to the criteria established by the DSM-IV TR), presence of cognitive impairment (Mini Mental State Exam scores ≥ 29), or dementia (assessed using the Blessed Information-Memory-Concentration Test, Blessed, 1996). These individuals ranged in age from 60 to 75 years of age (*M* = 66.92, *SD* = 5.02). Approximately half (51%) of this sample was male.

### 1.3 Procedure

#### 1.3.1 Sample of mothers and infants

Both the home and laboratory visits occurred between 900 and 1000 h. Infant afternoon cortisol levels are confounded by variations in daytime routine (Goldberg et al., 2003, Gunnar and White, 2001). Thus, the design used here is more sensitive to increases in infant than in maternal cortisol levels. It is important to note that it is unlikely that these samples are confounded by the cortisol awakening response (CAR), as the study occurred well after participants awoke. During the home visit, mothers and infants were observed in a Toy Frustration Task (TFT; Braungart-Rieker and Stifter, 1996). Approximately one month later, the dyads participated in the laboratory Strange Situation Procedure (SSP; Ainsworth et al., 1978). Saliva samples were collected from mothers and infants once before and twice after each task. Both procedures were discontinued if the infant cried continuously for 20 s.

#### 1.3.2 Sample of depressed adults

Individuals participated in two study sessions, the first consisting of a Structured Clinical Interview for DSM-IV, the second consisting of the Trier Social Stress Test (TSST; Kirschbaum et al., 1993). All TSST visits occurred between 1400 and 1840 h. Saliva samples were collected three times before and four times after the TSST. A complete description of study procedures are provided elsewhere (Chopra et al., 2009).

#### 1.3.3 Sample of older adults

The older adult sample was exposed to the TSST, which occurred between 1300 and 1800 h. Saliva samples were collected twice before and six times after the TSST. A complete description of similar study procedures can be found here (Sindi et al., 2013).

### 1.4 Measures

#### 1.4.1 Challenges for mothers and infants

The TFT (Braungart-Rieker and Stifter, 1996) consisted of four 90-s episodes: 1) Mother engages the infant with an attractive toy; 2) mother places the toy in a clear container with lid on but not sealed, while disengaging from the infant; 3) mother returns the toy to the infant; 4) mother places the toy in the clear container with the lid sealed and disengages again.

The SSP (Ainsworth et al., 1978) consisted of seven 3-min episodes, designed to induce increasing distress in the infant. During these episodes, 1) the infant and mother interact in an unfamiliar but child-friendly room, 2) a female stranger enters and engages the infant, 3) the mother leaves, 4) the mother returns and the female stranger leaves, 5) the mother leaves the infant alone, 6) the stranger returns, and 7) the mother returns and the stranger leaves. In this study, the SSP was used only as a stressor and was not coded for attachment classification (Ainsworth et al., 1978).

Due to ethical constraints, infant challenge paradigms are not highly stressful. Even so, among laboratory stressors, frustration paradigms (e.g., the TFT) are less potent than separation paradigms (e.g., the SSP). Based on meta-analytic data (Jansen et al., 2010), infant cortisol response to frustration paradigms corresponds to *d* (standardized difference between pre-stressor and post-stressor cortisol concentrations) = .19, whereas separation paradigms have an average effect of .34. Similar findings are reported in subsequent primary studies (Atkinson et al., 2013, Laurent et al., 2012). In addition, the TFT and SSP are differentially challenging for mothers and infants (Atkinson et al., 2013). The variable strength of these challenges is an important consideration, given the present focus on the meaning of cortisol indices, independent of stressor.

#### 1.4.2 Challenge for the depressed sample

Individuals completed the Trier Social Stress Task (Kirschbaum et al., 1993) in a standard interview room, which consisted of a video camera, microphone and stand, as well as a three-person “expert committee” who sat behind a table. Participants were given 10 min to prepare a 5-min speech (as if they were in a job interview); this preparation occurred in a separate room. After completing the speech, participants completed a serial subtraction mathematical problem. Salivary cortisol samples were obtained at times seven times throughout the study session.

#### 1.4.3 Challenge for the older adult sample

Similar to the sample of depressed participants, older adults completed the standard TSST in an interview room with a two-person (mixed gender) expert committee. Participants were given 10 min to prepare their speech. Participants were then introduced to the panel of experts. The experimenter sat behind a one-way mirror to observe participants complete the 5-min speech and 5-min mental arithmetic task. In total, eight saliva samples were collected before and after the TSST.

### 1.5 Salivary cortisol

#### 1.5.1 Sample of mothers and infants

Salivary cortisol was obtained from infants and mothers 5 min before each challenge and 20 and 40 min post-challenge. Two Sorbettes (Salimetrics, State College PA) were collected for each participant at each time point. Saliva samples were centrifuged for 10-min at 3000 rpm to extract the saliva and then stored in a freezer at −70 °C. Salivettes were thawed and centrifuged for 10 min at 3000 rpm at 4 °C. All samples were assayed twice using a salivary cortisol enzyme immunoassay kit (Salimetrics, State College, PA), and average values were used in analyses. The interassay variability was 10.6%; the intraassay variation was 8.3%, for samples with low values, and 6.9% for samples with high values.

#### 1.5.2 Sample of depressed adults

Salivary cortisol was obtained from participants two times before the TSST began (−35 min, −20 min), just before beginning the task (0 min), and four times after the task (+20, +40, +60, and +80 min). Saliva samples were collected using cotton wool salivettes (Sarstedt, Montreal, Quebec). All samples were assayed twice using radioimmunoassay kits (ICN Biomedical Inc, Costa Mesa, CA). Both intra- and inter-assay variability was less than 10%. For the purposes of this study, we used the cortisol samples collected at 0, +20 and +40.

#### 1.5.3 Sample of older adults

Salivary cortisol was obtained from participants 15 min before the beginning of the TSST (- 15 min), immediately before beginning the task (−1 min), and six times after the task (+1, +10, +20, +30, +45, and +60 min). Saliva samples were collected using salivettes (Sarstedt, Quebec City, Quebec, Canada). All samples were analyzed with a fluorescence immunoassay. Both intra- and inter-assay variability was less than 10%. For the purposes of this study, we used the cortisol samples collected at 0, +20 and +45, given that these time points closely resemble those of the primary samples.

#### 1.5.4 Cortisol indices

We used the baseline, +20 and +40^1^ times points in all samples to compute the fifteen cortisol indices identified in our literature survey. These time points were selected because they are consistent across the samples used here and are typically used in the literature. The fifteen cortisol indices include cortisol concentrations at each time point (i.e., baseline, 20, and 40 min), mean cortisol concentration, AUC_G_, AUC_I_, peak cortisol, minimum cortisol, cortisol reactivity, cortisol slope, percent change in cortisol, intercept and slope based on a regression of raw cortisol values. A description of all indices as well as the formulas used to derive them is included in Table 1.

### 1.6 Statistical analyses

Data analysis involved two phases. 1) We constructed correlation matrices for each sample, incorporating all cortisol indices and eliminating multicollinear indices. We then assessed the matrices for biometric adequacy. 2) We subjected the reduced matrices to principal components analysis.

#### 1.6.1 Correlation analyses

We conducted Pearson product–moment correlations, incorporating all 15 indices into the matrices—one for each sample (mother, infant, and depressed) and challenge (TFT, SSP, TSST). We examined correlation matrices for adequacy. Variables were excluded from further analyses if they correlated above .90 with at least two other variables across both participant (mothers and infants) and visit (home and lab). A .90 correlation or above indicates multicollinearity (Tabachnick and Fidell, 2007). The primary sample of mothers and infants were used to determine issues of multicollinearity, though similar multicollinear associations were also found in the depressed sample. Once all such variables were removed, we assessed it using Bartlett's test of sphericity (Bartlett, 1950) to ensure that variables were sufficiently intercorrelated, and the Kaiser-Meyer-Olkin measure of sampling adequacy (KMO), which assesses the degree to which each variable is explained by the others (Kaiser, 1974). These indices ensure that the matrices are sufficiently integrated to permit valid factor analysis.

#### 1.6.2 Principal component analyses (PCA)

Once matrix adequacy was established, six separate PCAs were conducted across participant and challenge. We used an oblique rotation (oblimin) to permit component dependence within solutions. Component loadings > .40 were interpreted (Field, 2009). Component retention was based on eigenvalue >1 (Guadagnoli and Velicer, 1988) and scree plot (Cattell, 1966) criteria. Tucker's coefficients of congruence (Tucker, 1951) were calculated to assess component congruence across participant (mothers, infants, depressed, and older adults) and time/challenge. Coefficients above .85 indicate high congruence, whereas coefficients above .95 indicate equivalent components (Lorenzo-Seva and Ten Berge, 2006).

An important strategy in labeling components involves a priori identification of marker variables whose meaning is known (Nunnally and Bernstein, 1994). We used AUC_G_ and AUC_I_ for this purpose, as they represent the two most-often used indices that capture cortisol levels across repeated measures. AUC_G_ measures total cortisol output, capturing both intensity (overall distance of cortisol samples from the ground) and sensitivity (difference between individual cortisol samples), whereas AUC_I_ measures change in cortisol over repeated samples, regardless of pre-challenge cortisol concentrations (Fekedulegn et al., 2007, Pruessner et al., 2003). Given the well-defined nature of these indices, as well as their popular use in the literature, we used AUC_G_ and AUC_I_ as our anchor points for defining the latent variables captured by the principal component analysis. This a priori expectation does not bias analyses but assists in the valid interpretation of extracted components.

## 2 Results

### 2.1 Descriptive statistics

Raw baseline, 20-min, and 40-min cortisol values were positively skewed (Table 2). Given that all cortisol indices were based on these values, all indices, except for the raw intercept and slope, were calculated and then log transformed to minimize skew. Table 2 displays the means and standard deviations for each index prior to transformation. Several indices have particularly large standard deviations, likely a product of intra- and interindividual variability in cortisol responses to challenge, a recognized phenomenon in the HPA literature (Atkinson et al., 2013, Kudielka et al., 2009). Several indices (i.e., maximum increase, slope, reactivity, peak reactivity, AUC_I_) remained non-normally distributed after transformation; however, log transformation minimized this skew and factor analysis is robust to violations of normality (Atkinson, 1988).

### 2.2 Correlation analyses

Correlation matrices (Table 3, Table 4, Table 5) revealed several variables with intercorrelations ≥ .90. This is not surprising, given that all variables are based on the same three values (baseline, 20-, and 40-min). This multicollinearity indicates that several variables do not explain unique variance beyond error. To render the matrix amenable to PCA, the following variables were excluded according to aforementioned multicollinearity criteria: mean, maximum increase, and peak cortisol values. The reactivity and raw slope variables correlated perfectly (*r* = 1.00) across all matrices, indicating that they explain identical variance. The reactivity variable was calculated by subtracting baseline from the 40-min value, whereas the slope variable was calculated using all three samples (baseline, 20 and 40 min). Given that the 20-min value correlates very highly with both the baseline and 40-min values (median *r* = .54) across all samples, it appears that the inclusion of 20-min values does not provide additional variance. Given that reactivity is more frequently used in the literature, the raw slope variable was excluded from subsequent analyses. Although all 15 variables are displayed in our correlation matrices (Table 3, Table 4, Table 5), these issues of multicollinearity resulted in the inclusion of 11 variables (baseline, 20-min, 40-min, minimum value, AUC_G_, AUC_I_, reactivity, peak reactivity, slope, intercept, and percent change) in subsequent analyses.

After excluding aforementioned variables, KMO values were adequate for all matrices, ranging from .52 to .68 for the primary sample of mothers and infants, .52 for the validation sample of depressed participants and .56 for the validation sample of older adults (Kaiser, 1974). Bartlett's test indicated no issues of sphericity for maternal (*X*^2^ (55) = 6361.84, *p* < .001; *X*^2^ (555) = 6288.65, *p* < .001), infant (*X*^2^ (55) = 4801.49, *p* < .001; *X*^2^ (55) = 5082.95, *p* < .001), depressed (*X*^2^ (55) = 1628.66, *p* < .001), or older adult (*X*^2^ (55) = 1442.20, *p* < .001) matrices.

### 2.3 Principal component analyses

#### 2.3.1 Mother and infant samples

Four PCAs were conducted (for mothers and infants separately, based on the TFT and SSP challenges). All analyses extracted a two-component solution. Component matrices are shown in Table 6, Table 7. For both mothers and infants, component 1 (with high loadings on baseline, 20 min, 40 min, minimum concentration, raw intercept, and, importantly, AUC_G_) appears to reflect *total cortisol production*. Component 2 (with high loadings on reactivity, peak reactivity, slope, percent change, and, importantly, AUC_I_) appears to reflect *change in cortisol* over time. In all instances the two-component solution accounts for a high percent of matrix variance (between 79% and 89%). At the home and laboratory visits, respectively, maternal component 1 accounted for 54.65% and 53.31% of the variance, and maternal component 2 accounted for 31.86% and 35.39% of the variance in cortisol indices. Similarly, for infants component 1 accounted for 44.53% and 48.30% of the variance, and component 2 accounted for 34.84% and 35.88% of the variance in cortisol indices at the home and laboratory visits, respectively. The high percent of variance explained by these components indicates that all cortisol indices are effectively captured by this bi-dimensional solution. Components 1 and 2 show low intercorrelations (maternal Components 1 and 2 correlate −.14 and −.082, for the TFT and SSP respectively; infant Components 1 and 2 correlate −.003 and .091 for the TFT and SSP respectively), indicating that components 1 and 2 are largely independent. Table 9 shows that component structure is stable across time for mothers and infants and congruent across mothers and infants during both the TFT and SSP. The factor analytic findings are robust.

#### 2.3.2 Clinically depressed sample

The component matrix for participants with depression is shown in Table 8. Similar to the mother and infant results, component 1 reflects *total cortisol production*, whereas component 2 reflects *change in cortisol* over time. Again, components 1 and 2 account for a high percent of the variance (93%). Components 1 and 2 are also largely independent, with an intercorrelation of .13. Three indices (i.e., 20 min, 40 min, and AUC_G_) have cross loadings in this validation sample, but not in the original sample. It is not entirely clear whether this is a substantive issue based on sample or challenge differences, or a statistical issue based on small sample size in this validation sample. As discussed below, however, these minor divergences have little influence on component congruence.

#### 2.3.3 Older adult sample

A PCA was also conducted for the sample of older adults (Table 8). Similar to the other PCA results, component 1 reflects *total cortisol output* and component 2 reflects *change in cortisol*. These two components account for 92% of the variance. Similar to the other samples, the intercorrelation between the two components was not significant at r = .13.

Table 9 shows that component structure is stable across participants and challenge. That is, coefficients of congruence ranged from .83 to .99 across participants during different challenges (e.g., infants and clinically depressed adults during the TFT and TSST, respectively). This is particularly notable given the small sample size of depressed and older adult samples.

## 3 Discussion

Cortisol is a commonly used marker of stress responsivity in both the developmental and adult literature. These literature incorporate an array of cortisol indices, often without clear definition or justification for use. Several investigators have suggested that this state of affairs is counterproductive (Atkinson et al., 2013, Dickerson and Kemeny, 2004). The present study examined associations amongst 15 cortisol indices (selected from a survey of recent HPA research), using primary samples of mother and infant cortisol collected during two challenges at two time points, as well as two validation samples of clinically depressed and healthy older adults. We conducted correlation and principal component analyses with these indices to explore their interrelationships and underlying dimensions and assist in bringing explicit biometric consensus to the literature.

To integrate and successfully interpret existing literature, a necessary first step is to understand how various cortisol indices are associated. Several variables were multicollinear, and there was wide variability in the correlation matrices of the remaining variables. The lower correlations were the first indication that these variables capture more than one dimension. At the same time, the higher correlations suggest that some variables share substantial variance; these results are a first indication that the plethora of indices utilized in the cortisol challenge literature is likely unnecessary, and superfluous.

Our next step was to examine what underlying dimensions are measured by the indices identified as nonredundant. All PCAs extracted two components, representing total cortisol production and change in cortisol over time (see Table 6, Table 7, Table 8). The components explained a very high percent of the variance (between 79 and 93 percent). In addition (and corollary to the high variance explained by the components themselves), the relevant component loadings are consistently very high. This indicates that each index is an effective marker of its underlying dimension. It also indicates, however, that many of these indices are redundant. Congruence coefficients showed that components are consistent across time, challenge and participant sample. Overall, the robust congruence coefficients attest to the reliability of the current findings.

Of the indices included in the PCA (keeping in mind that some indices were excluded prior to conducting the PCA), the AUC_G_ and AUC_I_ consistently loaded highly on component 1 and component 2, respectively, and each index loaded only weakly on the other component, across all samples. In addition, the reactivity and slope variables loaded very highly on component 2, and weakly on component 1, suggesting that they too could serve as potential markers for cortisol change. Overall, there is a need for consistent use of indices in this literature, with justification for index choice; the use of numerous indices measuring the same underlying construct (total cortisol or cortisol change) may thus be unnecessary and potentially confusing.

The demonstration of two largely independent components also underscores the need to determine whether these statistically derived constructs are related to physiologically distinct processes. To our knowledge, researchers have yet to directly explore whether there is a biological basis for the differentiation between total cortisol output and change in cortisol. Variability in cortisol reactivity is influenced by both genetic and environmental factors (Laurent et al., 2011, Young and Nolen-Hoeksema, 2001). However, research has not yet distinguished aspects of this cortisol responsivity. Similar questions have been explored in the diurnal cortisol literature. For example, the cortisol awakening response (CAR) is a discrete component of the cortisol diurnal cycle, with notable differences between the awakening response (measured as AUC) and mean cortisol levels throughout the day (Clow et al., 2004, Edwards et al., 2001). Further research demonstrates unique biological associations, such as genetics (Wust et al., 2000), neurobiology (Sage et al., 2001) and physiology (Pruessner et al., 1997) with CAR, but not daily circadian cortisol levels. These associations have yet to be explored in regards to differentiating cortisol responsivity to challenge. Our findings of two independent responsivity dimensions present a beginning point for investigation of differential physiological underpinnings.

In the present study we used diverse samples across different challenges. Interestingly, although the chronically depressed sample and the older adult sample both participated in the TSST, the chronic depressed sample had higher cortisol production compared to the healthy controls. In the literature, there is great variability in cortisol responses to different laboratory stressors (including the TSST), for depressed and older adult samples. The cortisol values reported in the literature for depressed and remitted depressed samples are sometimes lower than those presented here (e.g., Ahrens et al., 2008, Morris and Rao, 2013, Stewart et al., 2013), however, there are some studies that report values comparable to ours. For instance, one study showed similar cortisol responses to the TSST in individuals with remitted depression (Bagley et al., 2011). Other studies reported similar baseline cortisol values in depressed inpatients (Croes et al., 1993) and undergraduate samples with high depression (Scarpa and Luscher, 2002), in the context of non-TSST stressors. Other studies examining older adults with and without depression, showed variable cortisol responses to the TSST, with some groups exhibiting comparable cortisol levels to those found here (Armbruster et al., 2011, Taylor et al., 2006). Despite the cortisol production differences between the depressed and older adult samples, the factor structures were replicated between all samples.

The presented analyses, and the studies they are based on, are not without limitations. First, although the cortisol collection time points (i.e., baseline, 20- and 40/45 min) likely allowed for the capture of baseline and peak (Kirschbaum and Hellhammer, 2000, Schwartz et al., 1998, Stroud et al., 2009), they likely did not permit examination of cortisol recovery (Goldberg et al., 2003, Stroud et al., 2009). Second and related, these results are limited by the cortisol collection time points (baseline, 20- and 40/45 min) typically used in the literature and may not generalize to various sampling times reported in other studies. Research demonstrates that examining a high frequency of cortisol samples allows for a more nuanced understanding of the stress responses (e.g., Engert et al., 2013). Thus, further research should include a wider range of samples when examining the biometric properties of the cortisol response. Third, some of the indices remained non-normally distributed after transformation, though factor analysis and PCA is robust to non-normality (Atkinson, 1988). Fourth, the current analyses did not include cortisol trajectories (Davis and Sandman, 2010, Laurent et al., 2011), so we cannot comment on their relation to the composite indices assessed here. A major strength of this study, one the other hand, involves the use of diverse samples, which vary across developmental stage, mental health status, and challenge, offering some assurance of generalizability.

## 4 Conclusion

In summary, PCA of 15 cortisol indices revealed a consistent two-component structure, representing total cortisol output and cortisol change. This component structure was reliable across time, challenge and participant. This study provides an early step in gaining biometric clarification of the cortisol response literature.

## Declaration of interests

The authors do not have any financial or personal interests to disclose.

## Figures and Tables

**Table 1:** Definitions of cortisol indices as identified in the literature search and calculated in the current samples.

Cortisol index	Number of times used in literature	Definition/formula for current samples
Baseline cortisol value	51	Cortisol value at baseline or pre-challenge
20 min cortisol value	41^a^	Cortisol value at 20 min post-challenge
40 min cortisol value	41^a^	Cortisol value at 40 min post-challenge
Mean cortisol	12	Average cortisol values across all samples. = Σ(baseline + 20 min + 40 min)/3
AUC_G_	19	Area under the curve with respect to ground. = {[(20 min value + baseline value)/2] x time} + {[(40 min value + 20 min value)/2] x time}
AUC_I_	9	Area under the curve with respect to increase (or change).^b^ = {[(20 min value + baseline value)/2] x time} + {[(40 min value + 20 min value)/2] x time} – [baseline value* (time + time)]
AUC_AB_	1	Area under the curve above/below baseline.^b^ = {[(20 min – baseline)/2]*time} + {[(20 min – baseline) + (40 min – baseline)/2]*time}
Peak	3	Highest cortisol value (either baseline, 20 min, or 40 min sample).
Minimum	1	Lowest cortisol value (either baseline, 20 min, or 40 min sample).
Maximum increase	1	Highest cortisol value minus lowest cortisol value.
Reactivity	10	Change in cortisol between the baseline and 40 min values. = 40 min – baseline
Peak reactivity	3	Change in cortisol between the baseline and peak values. = peak (either baseline, 20 min, or 40 min) – baseline
Slope	3	Slope of the line between baseline and 40 min cortisol value. = (40 min – baseline)/time.
Regression intercept (raw)	4	Intercept of the regression line fitted through the raw cortisol data.
Regression slope (raw)	3	Slope of the regression line fitted through the raw cortisol data.
Percent change	1	Percent change between the first and last cortisol collection. = ((40 min – baseline)/(baseline)*100)

*Note*. Time values represent the time between cortisol samples, which in the current analyses equals 20 min. AUC_G_ = area under the curve with respect to ground; AUC_I_ = area under the curve with respect to increase; AUC_AB_ = area under the curve above baseline.

a Forty one of the studies in the literature search used individual time points of cortisol collection, however, these time points were not necessarily 20 and 40 min.

b As evident from the formulas, AUC_I_ and AUC_AB_ are identical indices, based on different but equivalent formula.

**Table 2:** Descriptive Statistics for raw cortisol values.

Cortisol index	Sample	TFT	SSP	Sample	TSST
Baseline cortisol value	Mothers	7.06 (4.32)	7.72 (5.94)	Depressed	14.37 (5.59)
Infants	5.23 (3.63)	4.92 (4.74)	Older adults	4.25 (3.15)
20 min cortisol value	Mothers	5.16 (4.30)	5.33 (3.93)	Depressed	18.17 (8.7)
Infants	4.14 (3.13)	6.67 (20.07)	Older adults	6.01 (4.12)
40 min cortisol value	Mothers	4.68 (3.07)	4.77 (3.81)	Depressed	16.78 (7.08)
Infants	4.39 (4.76)	8.31 (30.62)	Older adults	5.74 (4.54)
Mean cortisol	Mothers	5.62 (2.89)	5.85 (3.72)	Depressed	16.44 (6.34)
Infants	4.48 (2.79)	5.25 (4.75)	Older adults	5.34 (3.33)
AUC_G_	Mothers	220.45 (115.99)	227.90 (143.91)	Depressed	675.09 (274.39)
Infants	175.82 (111.51)	211.67 (186.85)	Older adults	220.28 (139.84)
AUC_I_	Mothers	−60.07 (99.11)	−80.34 (156.76)	Depressed	100.10 (184.66)
Infants	−31.84 (92.38)	15.49 (127.18)	Older adults	50.21 (117.16)
Peak	Mothers	7.66 (4.47)	8.21 (6.32)	Depressed	20.09 (8.17)
Infants	6.11 (4.19)	7.52 (6.47)	Older adults	7.30 (5.18)
Minimum	Mothers	4.10 (2.07)	4.14 (2.72)	Depressed	12.85 (5.36)
Infants	3.10 (1.74)	3.44 (3.00)	Older adults	3.45 (2.03)
Maximum increase	Mothers	7.00 (4.29)	7.71 (6.15)	Depressed	7.06 (5.83)
Infants	7.02 (7.81)	5.67 (4.15)	Older adults	3.77 (4.19)
Reactivity	Mothers	3.10 (1.74)	3.44 (3.00)	Depressed	2.41 (6.35)
Infants	−.98 (3.83)	.59 (5.60)	Older adults	1.49 (4.77)
Peak reactivity	Mothers	.53 (2.22)	.51 (2.21)	Depressed	5.61 (6.50)
Infants	.72 (2.71)	2.41 (5.24)	Older adults	3.00 (4.09)
Slope	Mothers	−.049 (.076)	−.047 (.079)	Depressed	3.61 (9.52)
Infants	−.0098 (.16)	.0090 (.087)	Older adults	1.99 (6.36)
Regression intercept (raw)	Mothers	6.82 (3.90)	7.40 (5.31)	Depressed	15.24 (6.13)
Infants	5.02 (3.40)	4.98 (4.50)	Older adults	4.65 (3.18)
Regression slope (raw)	Mothers	−.061 (.086)	−.078 (.13)	Depressed	.060(.16)
Infants	−.027 (.088)	.014 (.14)	Older adults	.031 (.10)
Percent change	Mothers	−25.35 (44.52)	−32.42 (37.63)	Depressed	26.72 (61.21)
Infants	−5.66 (69.83)	39.46 (123.02)	Older adults	60.69 (129.86)

*Note*. Mean (standard deviation); TFT = Toy Frustration Task; SSP = Strange Situation Procedure; TSST = Trier Social Stress Test; Baseline = baseline cortisol value; 20 min = cortisol value at 20 min post-challenge; 40 min = cortisol value at 40 min post-challenge; Mean = average cortisol value; AUC_G_ = area under the curve with respect to ground; AUC_I_ = area under the curve with respect to increase; Peak = peak cortisol value; Minimum = minimum cortisol value; Maximum increase = peak value minus minimum value; Reactivity = 40-min sample minus baseline; Peak reactivity = peak sample minus baseline sample; Slope = slope from baseline to 40 min value; Intercept = intercept of the regression line fitted through the raw cortisol data; Regression slope = slope of the regression line fitted through the raw cortisol data; Percent change = percent increase/decrease from 0 to 40/45 min.

**Table 3:** Correlation matrix of maternal cortisol variables during the toy frustration and strange situation procedures.

	1. Baseline	2. 20 min	3. 40 min	4. Mean	5. AUC_G_	6. AUC_I_	7. Pk	8. Min	9. Max	10. RT	11. PkRT	12. Slp	13. Int	14. Slp_raw_	15. % change
1.	*.37*	.73	.69	.90	.86	−.67	.88	.80	.90	−.66	−.12	−.56	.93	−.69	−.36
2.	.69	*.31*	.81	.93	.96	−.11	.85	.87	.85	−.24	.36	−.26	.77	−.24	.092
3.	.67	.86	*.36*	.89	.88	−.12	.76	.91	.78	−.02	.30	−.042	.65	−.04	.35
4.	.90	.91	.89	*.40*	.99	−.36	.97	.91	.96	−.38	.23	−.366	.88	−.38	.017
5.	.87	.94	.89	.99	*.39*	−.30	.96	.91	.95	−.35	.28	−.34	.86	−.35	.040
6.	−.65	−.058	−.089	−.35	−.28	*.087*	−.43	−.27	−.44	.92	.55	.76	−.68	.92	.64
7.	.92	.75	.71	.96	.95	−.47	*.23*	.79	.99	−.46	.30	−.42	.89	−.46	−.029
8.	.75	.89	.96	.90	.91	−.17	.77	*.46*	.77	−.27	.044	−.25	.76	−.27	.021
9.	.93	.79	.75	.95	.94	−.49	.99	.75	*.32*	−.47	.29	−.43	.89	−.47	−.036
10.	−.61	−.081	−.018	−.37	−.32	.93	−.48	−.10	−.50	*.086*	.50	.84	−.73	1.00	.77
11.	−.065	.41	.31	.27	.31	.47	.30	.18	.28	.34	*.19*	.38	−.006	.50	.70
12.	−.62	−.10	−.025	−.38	−.33	.91	−.48	−.12	−.50	.99	.35	*.10*	−.64	.84	.62
13.	.88	.61	.58	.82	.80	−.63	.86	.62	.87	−.77	.067	−.77	*.24*	−.73	−.300
14.	−.62	−.081	−.016	−.37	−.32	.93	−.49	−.10	−.50	1.00	.35	.99	−.77	*.073*	.77
15.	−.33	.23	.40	.063	.10	.62	−.045	.19	−.064	.58	.65	.60	−.23	.59	*.094*

*Note*: Correlations above the diagonal (shaded grey) refer to the Toy Frustration task, correlations below the diagonal refer to the Strange Situation procedure. Diagonal values (italicized, underlined) are test–retest correlations (across stressors). Correlations above .18 and .13 are significant at p < .01 and p < .05, respectively. Baseline = baseline cortisol value; 20-min = cortisol value at 20 min post-challenge; 40-min = cortisol value at 40 min post-challenge; mean = average cortisol value across samples; Pk = peak cortisol value; Min = minimum cortisol value; Max = maximum increase (peak minus minimum value); AUC_G_ = area under the curve with respect to ground; AUC_I_ = area under the curve with respect to increase; PkRT = peak reactivity; Slp = slope from baseline to 40 min value; Int = intercept of the regression line fitted through the raw cortisol data; Slp_Raw_ = slope of the regression line fitted through the raw cortisol data; % change = percent increase/decrease from 0 to 40 min.

**Table 4:** Correlation matrix of infant cortisol variables during the toy frustration and strange situation procedures.

	1. Baseline	2. 20 min	3. 40 min	4. Mean	5. AUC_G_	6. AUC_I_	7. Pk	8. Min	9. Max	10. RT	12. PkRT	12. Slp	13. Int	14. Slp_raw_	15. % change
1.	*.37*	.61	.54	.94	.80	−.61	.73	.72	.84	−.42	−.13	−.073	.89	−.52	−.35
2.	.56	*.16*	.74	.89	.93	.11	.73	.85	.79	−.007	.42	−.023	.66	−.005	.17
3.	.45	.76	*.34*	.86	.85	.084	.57	.83	.76	.37	.38	.15	.50	.31	.50
4.	.76	.90	.85	*.28*	.99	−.20	.97	.88	.95	−.11	.38	.026	.81	−.11	.11
5.	.72	.95	.83	.99	*.26*	−.13	.95	.89	.93	−.086	.41	.019	.79	−.084	.13
6.	−.42	.28	.30	.084	.15	*−.047*	−.28	−.067	−.29	.81	.59	.085	−.57	.81	.63
7.	.65	.77	.63	.97	.97	.071	*.039*	.76	.99	−.23	.49	.016	.82	−.17	.076
8.	.78	.76	.78	.85	.84	−.006	.74	*.21*	.72	−.025	.22	−.029	.65	−.023	.045
9.	.71	.85	.78	.96	.96	.073	.99	.71	*.26*	−.18	.42	.054	.83	−.18	.11
10.	−.24	.18	.53	.21	.21	.75	.19	.11	.21	*.074*	.43	.29	−.58	1.00	.77
11.	.12	.56	.62	.60	.62	.60	.67	.36	.66	.74	*.018*	.19	.031	.67	.64
12.	−.23	.19	.54	.23	.23	.74	.20	.12	.23	.99	.75	*.074*	−.10	.24	.31
13.	.86	.67	.47	.76	.76	−.36	.76	.72	.76	−.23	.30	−.21	*.24*	−.57	−.29
14.	−.24	.18	.52	.22	.22	.74	.22	.11	.22	1.00	.76	.99	−.22	*.084*	.79
15.	−.36	.16	.51	.20	.21	.53	.25	−.075	.28	.72	.60	.72	−.27	.72	*.062*

*Note*: Correlations above the diagonal (shaded grey) refer to the Toy Frustration task, correlations below the diagonal refer to the Strange Situation procedure. Diagonal values (italicized, underlined) are test–retest correlations (across stressors). Correlations above .18 and .13 are significant at p < .01 and p < .05, respectively. Baseline = baseline cortisol value; 20-min = cortisol value at 20 min post-challenge; 40-min = cortisol value at 40 min post-challenge; mean = average cortisol value across samples; Pk = peak cortisol value; Min = minimum cortisol value; Max = maximum increase (peak minus minimum value); AUC_G_ = area under the curve with respect to ground; AUC_I_ = area under the curve with respect to increase; RT = reactivity; PkRT = peak reactivity; Slp = slope from baseline to 40 min value; Int = intercept of the regression line fitted through the raw cortisol data; Slp_Raw_ = slope of the regression line fitted through the raw cortisol data; % change = percent increase/decrease from 0 to 40 min.

**Table 5:** Correlation matrix of depressed and older adult participant's cortisol variables during the Trier Social Stress Test.

	1. baseline	2. 20 min	3. 40 min	4. Mean	5. AUC_G_	6. AUC_I_	7. Pk	8. Min	9. Max	10. RT	11. PkRT	12. Slp	13. Int	14. Slp_raw_	15. % change
1.	*–*	.63	.58	.79	.75	−.19	.74	.89	.34	−.21	.080	−.24	.86	−.21	−.28
2.	.69	*–*	.85	.94	.97	.48	.94	.82	.67	.36	.65	.33	.69	.35	.34
3.	.49	.75	*–*	.93	.92	.57	.92	.72	.71	.59	.75	.57	.51	.59	.55
4.	.79	.94	.87	*–*	.99	.36	.98	.86	.70	.33	.62	.30	.75	.33	.32
5.	.77	.97	.85	.99	*–*	.40	.98	.86	.70	.34	.63	.31	.74	.33	.33
6.	−.13	.554	.64	.47	.50	*–*	.37	.15	.40	.93	.82	.95	−.24	.92	.73
7.	.65	.97	.84	.97	.97	.58	*–*	.78	.79	.35	.68	.31	.73	.34	.37
8.	.84	.84	.71	.87	.86	.21	.71	*–*	.32	.056	.26	.043	.76	.046	−1.1
9.	.086	.63	.51	.55	.58	.74	.71	.089	*–*	.51	.85	.44	.48	.51	.66
10.	−.30	.26	.62	.28	.28	.86	.40	.015	.62	*–*	.86	.99	−.28	1.00	.84
11.	−.059	.65	.64	.55	.58	.94	.67	.17	.92	.82	*–*	.83	.12	.86	.83
12.	−.30	.26	.62	.27	.27	.86	.38	.024	.58	.99	.81	*–*	−.33	.99	.82
13.	.94	.79	.56	.85	.84	.078	.76	.84	.29	−.19	.17	−.20	*–*	−.29	−.18
14.	−.30	.26	.62	.28	.28	.86	.40	.015	.62	1.00	.83	.99	−.19	*–*	.85
15.	−.47	.11	.46	.12	.12	.72	.29	−.20	.55	.89	.70	.88	−.34	.89	*–*

*Note*: Correlations below the diagonal are for the depressed sample, correlations above the diagonal are for the older adult sample. Correlations above .28 and .43 are significant at p < .01 and p < .05, respectively. Baseline = baseline cortisol value; 20-min = cortisol value at 20 min post-challenge; 40-min = cortisol value at 40 min post-challenge; mean = average cortisol value across samples; Pk = peak cortisol value; Min = minimum cortisol value; Max = maximum increase (peak minus minimum value); AUC_G_ = area under the curve with respect to ground; AUC_I_ = area under the curve with respect to increase; RT = reactivity; PkRT = peak reactivity; Slp = slope from baseline to 40 min value; Int = intercept of the regression line fitted through the raw cortisol data; Slp_Raw_ = slope of the regression line fitted through the raw cortisol data; % change = percent increase/decrease from 0 to 40 min.

**Table 6:** Component matrices for maternal cortisol indices during the toy frustration and strange situation challenges, Principal Components Extraction, oblimin rotation.

Cortisol variable	TFT (time 1; *N* = 258)		SSP (time 2; *N* = 231)	
Component 1	Component 2	Component 1	Component 2
Baseline	**.86**	−**.57**	**.85**	−**.55**
20 min	**.94**	−.023	**.94**	.052
40 min	**.91**	.12	**.94**	.15
Minimum	**.93**	−.14	**.94**	−.019
AUC_G_	**.99**	−.15	**.99**	−.12
AUC_I_	−.31	**.91**	−.28	**.91**
RT	−.34	**.95**	−.27	**.95**
PkRT	.27	**.71**	.34	**.66**
Slp	−.33	**.83**	−.27	**.95**
Int	**.87**	−**.55**	**.87**	−**.49**
% change	.072	**.88**	.17	**.87**

*Note*. Only component loadings > 0.4 are bolded. TFT = Toy Frustration Task; SSP= Strange Situation Procedure; Baseline = baseline cortisol value; 20 min = cortisol value at 20 min post-challenge; 40 min = cortisol value at 40 min post-challenge; Minimum = minimum cortisol value; AUC_G_ = area under the curve with respect to ground; AUC_I_ = area under the curve with respect to increase; RT = reactivity; PkRT = peak reactivity; Slp = slope from baseline to 40 min value; Int = intercept of the regression line fitted through the raw cortisol data; % change = percent increase/decrease from 0 to 40 min.

**Table 7:** Component loadings for infant cortisol indices during the toy frustration and strange situation challenges, using oblimin rotation.

Cortisol variable	TFT (time 1; *N* = 239)		SSP (time 2; *N* = 208)	
Component 1	Component 2	Component 1	Component 2
Baseline	**.82**	−**.50**	**.85**	−**.40**
20 min	**.91**	.16	**.87**	.29
40 min	**.84**	.39	**.78**	**.56**
Minimum	**.91**	.035	**.92**	.084
AUC_G_	**.99**	.047	**.96**	.28
AUC_I_	−.16	**.86**	−.043	**.85**
RT	−.12	**.94**	.077	**.95**
PkRT	.38	**.79**	**.50**	**.80**
Slp	.004	.33	.088	**.95**
Int	**.82**	−**.46**	**.88**	−.24
% change	.091	**.90**	.003	**.83**

*Note*. Only component loadings greater than 0.4 are bolded. TFT = Toy Frustration Task; SSP= Strange Situation Procedure; Baseline = baseline cortisol value; 20 min = cortisol value at 20 min post-challenge; 40 min = cortisol value at 40 min post-challenge; Min = minimum cortisol value; AUC_G_ = area under the curve with respect to ground; AUC_I_ = area under the curve with respect to increase; RT = reactivity; PkRT = peak reactivity; Slp = slope from baseline to 40 min value; Int = intercept of the regression line fitted through the raw cortisol data; % change = percent increase/decrease from 0 to 40 min.

**Table 8:** Component loadings for cortisol indices of depressed and older adult individuals during the Trier Social Stress Task, using oblimin rotation.

Cortisol variable	Depressed (*N* = 48)		Older adults (*N* = 51)	
Component 1	Component 2	Component 1	Component 2
Baseline	**.90**	−.28	**.96**	−.30
20 min	**.89**	**.50**	**.85**	.36
40 min/45 min	**.74**	**.69**	**.73**	**.56**
Minimum	**.93**	.10	**.95**	−.034
AUC_G_	**.93**	**.45**	**.91**	.31
AUC_I_	.27	**.95**	−.009	**.95**
RT	.050	**.98**	−.068	**.98**
PkRT	.31	**.90**	.27	**.88**
Slp	.050	**.98**	−.10	**.98**
Int	**.95**	−.092	**.93**	−.33
% change	−.12	**.91**	−.093	**.91**

*Note*. Only component loadings greater than 0.4 are bolded. Baseline = baseline cortisol value; 20 min = cortisol value at 20 min post-challenge; 40 min = cortisol value at 40 min post-challenge; Min = minimum cortisol value; AUC_G_ = area under the curve with respect to ground; AUC_I_ = area under the curve with respect to increase; RT = reactivity; PkRT = peak reactivity; Slp = slope from baseline to 40 min value; Int = intercept of the regression line fitted through the raw cortisol data; % change = percent increase/decrease from 0 to 40 min.

**Table 9:** Tucker's coefficients of congruence.

	Component 1	Component 2
Infant TFT and SSP	.99	.94
Mother TFT and SSP	.98	.99
Infant and mother (TFT)	.98	.95
Infant and mother (SSP)	.92	.94
Infant (TFT) and depressed (TSST)	.97	.91
Infant (SSP) and depressed (TSST)	.98	.99
Mother (TFT) and depressed (TSST)	.93	.87
Mother (SSP) and depressed (TSST)	.87	.89
Infant (TFT) and older adult (TSST)	.83	.88
Infant (SSP) and older adult (TSST)	.99	.99
Mother (TFT) and older adult (TSST)	.97	.93
Mother (SSP) and older adult (TSST)	.93	.94
Older adult (TSST) and depressed (TSST)	.99	.99

## Footnotes

1 The + 45 min sample was used for the older adult sample, as saliva was not collected at +40 min. Throughout the remainder of the paper, reference to the 40 min samples includes the 45 min sample for older adults.
